# Evaluating Cetuximab Regimens in Head and Neck Cancer: Insights from a Retrospective Cohort Study

**DOI:** 10.3390/cancers17020210

**Published:** 2025-01-10

**Authors:** Chih-Chun Wang, Shyh-An Yeh, Wen-Hui Chen, Hung-Ju Li, Chuan-Chien Yang, Tse-Jen Huang, Yu-Chieh Su

**Affiliations:** 1 Department of Otolaryngology, E-Da Hospital, I-Shou University, Kaohsiung 82445, Taiwan; cw5969@yahoo.com.tw (C.-C.W.); edah620901@yahoo.com.tw (C.-C.Y.); ed103548@edah.org.tw (T.-J.H.); 2 School of Chinese Medicine for Post-Baccalaureate, College of Medicine, I-Shou University, Kaohsiung 82445, Taiwan; 3 Department of Radiation Oncology, E-Da Hospital, I-Shou University, Kaohsiung 82445, Taiwan; sayeh@outlook.com; 4 Department of Medical Imaging and Radiological Sciences, I-Shou University, Kaohsiung 82445, Taiwan; 5 Department of Dentistry, E-Da Hospital, I-Shou University, Kaohsiung 82445, Taiwan; wenhuichen0802@gmail.com; 6 School of Medicine for International Students, College of Medicine, I-Shou University, Kaohsiung 82445, Taiwan; 7 Division of Hematology-Oncology, Department of Internal Medicine, E-Da Hospital, I-Shou University, Kaohsiung 82445, Taiwan; ciepur@gmail.com; 8 School of Medicine, College of Medicine, I-Shou University, Kaohsiung 82445, Taiwan; 9 Graduate Institute of Medicine, College of Medicine, I-Shou University, Kaohsiung 82445, Taiwan

**Keywords:** head and neck cancer, cetuximab, recurrence, metastasis, retrospective study

## Abstract

Background/Objectives: The aim of this study is to assess the effectiveness of cetuximab combination therapy in patients with recurrent or metastatic head and neck cancer treated at a hospital in Southern Taiwan. Methods: This study analyzed a retrospective cohort of 67 patients who were treated between January 2020 and May 2024 with two cetuximab regimens, cetuximab combined with cisplatin and 5-Fu, which were administered every four weeks during hospitalization (CPF4) and every two weeks as outpatient treatment (CPF2), respectively. The clinical outcomes, including overall survival and progression-free survival (PFS), were compared across the treatment regimens and age groups using Kaplan–Meier survival curves and Cox proportional hazard models. Results: The median overall survival was 11.1 months (95% confidence interval, 7.8–14.5), with CPF2 showing a potential PFS advantage in patients aged 46–60 years (*p* = 0.049). No significant differences in overall survival were observed between CPF2 and CPF4. CPF2, which was administered in an outpatient setting, was associated with improved convenience, reduced hospitalization, and potentially lower risks of hospital-acquired infections. Conclusions: CPF2 exhibits practical advantages and comparable effectiveness, making it the preferred treatment regimen for eligible patients. Further studies with larger populations and molecular stratifications are needed to confirm these findings and develop better treatment strategies.

## Simple Summary

This study investigates the outcomes of cetuximab combination therapy in recurrent or metastatic head and neck cancer patients treated at a hospital in Southern Taiwan. A total of 67 patients were retrospectively analyzed, comparing two treatment regimens: CPF4, administered every four weeks during hospitalization, and CPF2, administered every two weeks as an outpatient procedure. The results showed a progression-free survival advantage for patients aged 46–60 years treated with CPF2, though no significant differences in overall survival were observed between CPF4 and CPF2 in any age group. CPF2 demonstrated practical benefits, including reduced hospitalization, lower infection risks, and improved patient convenience, making it a preferred choice for certain patients. These findings highlight the potential impact of age on treatment responses and the importance of balancing clinical outcomes with patient quality of life. Further large-scale studies are required to confirm these observations and refine treatment strategies.

## 1. Introduction

Head and neck cancers typically originate from squamous cells lining the mucosal surfaces of the head and neck as well as the salivary glands, sinuses, or muscles and nerves of the head and neck. Based on the primary site of origin, they can be classified as follows: oral cancer, oropharyngeal cancer, hypopharyngeal cancer, laryngeal cancer, and nasopharyngeal cancer. Recent data have indicated that in 2022, head and neck cancer was the sixth most common cancer globally, accounting for approximately 946,000 new cases (4.7% of all new cases) and 482,000 deaths (5.0% of all cancer deaths) [1].

In Taiwan, head and neck cancer ranks among the top five causes of cancer-related deaths [2]. According to the 2021 Cancer Registry Annual Report from the Health Promotion Administration, Ministry of Health and Welfare, Taiwan, approximately 10,800 new cases of head and neck cancer, accounting for 8.8% of all new cancer cases, were recorded. Patients with primary sites in the oral cavity, oropharynx, and hypopharynx comprised the majority of all cases (three-quarters of all head and neck cancers), with approximately 8200 cases. The crude incidence rate translates to 35 cases of oral cancer per 100,000 people, making it the sixth most common cancer in Taiwan. Moreover, the crude incidence rate among Taiwanese men is 64 cases per 100,000 people, which is approximately nine times higher than that of Taiwanese women, making it the third most common type of cancer among men in Taiwan [2]. The age-standardized incidence rate of oral cancer among Taiwanese men in 2021 (40.4 per 100,000 people) was 1.5 times higher than that in 2001 (26.9 per 100,000 people), while the number of men dying from oral cancer also increased from 1436 deaths in 2001 (age-standardized mortality rate of 12.03 per 100,000 people) to 2463 deaths in 2011 (age-standardized mortality rate of 15.06) and 3110 deaths in 2021 (age-standardized mortality rate of 16.38 [3,4]). This suggests the need for more aggressive and effective drugs or treatment regimens for squamous cell carcinoma of the head and neck (SCCHN).

In addition to traditional surgical and radiation therapies, treatments for head and neck cancers also include chemotherapy, concurrent chemoradiotherapy (CCRT), targeted therapies, and even immunotherapy [5]. Radiotherapy, often used as a primary treatment or in combination with systemic therapies, remains a cornerstone in managing locally advanced head and neck cancers. Recent advances have integrated radiotherapy with systemic agents, including chemotherapy, cetuximab, and emerging immunotherapies, improving survival and reducing toxicity [6]. Furthermore, studies have highlighted complications such as oral mucositis and their impact on patient outcomes during radiotherapy [7]. The most important target for treatment strategies in head and neck cancers is the epidermal growth factor receptor (EGFR). Cetuximab, a monoclonal antibody targeting EGFR, was approved by the US Food and Drug Administration in 2006 as a treatment for SCCHN. It can be combined with radiotherapy and has a significant effect on locally invasive head and neck cancers and can also be used as a single drug to treat recurrent or metastatic head and neck cancers [8]. In Taiwan, under the guidelines of the National Health Insurance system, cetuximab is the primary drug used for treating metastatic and recurrent head and neck cancers. The treatment regimens include cetuximab monotherapy, cetuximab combined with the use of chemotherapy drugs such as cisplatin and fluorouracil (5-Fu) with radiation therapy, and cetuximab combined with docetaxel.

Although immunotherapy combined with chemotherapy has been shown to provide superior efficacy compared to cetuximab-based regimens, with anti-PD-1 antibodies such as pembrolizumab and nivolumab significantly improving survival rates and quality of life in head and neck cancer patients [9,10,11,12], cetuximab plus chemotherapy remains a valuable option for patients who are not eligible for immunotherapy. This highlights the importance of optimizing cetuximab-based treatment regimens for specific patient populations.

This study aims to analyze the survival rate of recurrent or metastatic head and neck cancer patients who received cetuximab combination therapy at a hospital in Southern Taiwan. Furthermore, the present study investigated whether different treatment regimens influence the survival of patients with head and neck cancers, with the goal of identifying the optimal treatment approach based on the trial outcomes.

## 2. Materials and Methods

### 2.1. Data Sources

This retrospective observational cohort study included patients with recurrent or metastatic head and neck cancers who underwent cetuximab treatment at E-Da Hospital, Taiwan, between 1 January 2020 and 31 May 2024, based on a review of medical records. The head and neck cancer types in this study were classified based on the International Classification of Diseases for Oncology, Third Edition (ICD-O-3) [13]. These included buccal cancer, cheek mucosa cancer, external lower lip cancer, floor of mouth cancer, hard palate cancer, lower lip cancer, oral cavity cancer, palate cancer, retromolar area cancer, upper gum cancer, soft palate cancer, tongue cancer, hypopharyngeal cancer, laryngeal cancer, oropharyngeal cancer, and lower gum cancer. A total of 96 medical records of patients who underwent cetuximab treatment were retrospectively reviewed (including only one female patient). After excluding the cases that were not administered cetuximab in the subsequent treatments or those who received cetuximab for less than 1 month, 67 cases met the inclusion criteria and were included in the analysis.

The acquired patient medical record data from the hospital included information on gender, age at diagnosis, disease diagnosis, date of initial diagnosis, primary tumor site, clinical stage, pathological stage, p16 status, date of first recurrence, date of recurrence at the time of cetuximab administration, disease extent (metastatic or not), cancer treatment history, time interval since the last use of cisplatin, cetuximab treatment method, the application of immune-oncology therapy (IO therapy), start and end dates of the cetuximab treatment, last follow-up date, and date of death. The reasons for treatment discontinuation were recorded and analyzed, with particular attention to adverse effects and their potential impact on treatment adherence. In this study, no patients discontinued or delayed treatment due to adverse effects. Treatment discontinuation was primarily attributed to disease progression rather than toxicity-related complications. All information regarding the primary cancer site and histology was coded using the ICD-O-3 classification system (ICD-O-3 codes: C00-C14, except for C07-C08 and C11) [13].

The patients were categorized by age into three groups: (1) 20–45 years, (2) 46–60 years, and (3) 61–75 years. These groupings were determined based on the dataset’s age distribution, which ranged from 28 to 74 years. Initially, 15-year intervals (30–45, 46–60, and 61–75) were considered. However, due to the youngest patient being 28 years old and the rarity of cases under 30 years in the general population, the first group was expanded to include patients aged 20–45 years. This adjustment ensured more representative and clinically meaningful stratification for analysis. The primary tumor sites were classified as (1) the oral cavity and (2) the pharynx. The disease extent was classified into three categories: (1) local recurrent, (2) distant metastatic, and (3) distant metastatic, including local recurrent. Based on the cancer treatment history, the patients were divided into two groups: (1) with operation (OP) and (2) CCRT without OP.

The treatment regimens were categorized into three groups:

(1) CPF4 regimen: Cetuximab combined with cisplatin and 5-Fu, administered every four weeks during hospitalization. Cetuximab is given at a dose of 400 mg/m^2^ on Days 1 and 14, cisplatin is administered at 70–90 mg/m^2^ on Day 1, and 5-Fu is delivered daily at a dose of 700–900 mg/m^2^ from Days 1 to 4.
(2) CPF2 regimen: Cetuximab combined with cisplatin and fluorouracil (5-Fu), administered every two weeks in an outpatient setting. In this regimen, cetuximab is administered at a dose of 400 mg/m^2^ on Day 1, cisplatin at 35–40 mg/m^2^ on Day 1, and 5-Fu at a total dose of 2000 mg/m^2^ over Days 1 and 2.
(3) CTPF regimen: Cetuximab combined with docetaxel, cisplatin, and fluorouracil (5-Fu), administered approximately every three to four weeks in either an outpatient or inpatient setting. Cetuximab is given at a dose of 400 mg/m^2^ on Day 1, docetaxel at 70 mg/m^2^ on Day 1, cisplatin at 70 mg/m^2^ on Day 1, and 5-Fu is delivered daily at a dose of 700 mg/m^2^ from Days 1 to 4.

The median cycle counts for CPF4, CPF2, and CTPF regimens were 3, 4, and 4, respectively. The corresponding median treatment durations were 8.1 weeks for CPF4, 18.4 weeks for CPF2, and 13.1 weeks for CTPF. While the cycle counts did not differ significantly among the three regimens (*p*-value = 0.056, Kruskal–Wallis test), a significant difference was observed in treatment durations (*p*-value = 0.023). Regarding hospitalization, patients in the CPF4 group had a mean hospital stay of 32.6 ± 15.4 days (median: 30 days; range: 6–69 days), reflecting the inpatient nature of this regimen. Similarly, patients in the CTPF group had a mean hospital stay of 29 ± 19.5 days (median: 28 days; range: 6–50 days), as this regimen was also frequently administered in an inpatient setting. In contrast, the CPF2 group, primarily an outpatient regimen, had an average inpatient stay of 6.2 ± 10.7 days (median: 1 day; range: 0–45 days), which was often due to complications or other medical needs during the treatment period.

### 2.2. Ethics Statement

The study protocol was reviewed and approved by the Institutional Review Board of E-Da Hospital (IRB number: EMRP-113-104) and implemented in accordance with the Declaration of Helsinki.

### 2.3. Outcomes of Interest

Overall survival and progression-free survival (PFS) were the outcomes of interest in this study. Overall survival was defined as the time from the initiation of cetuximab treatment to the date of last contact with the patient or death from any cause, whereas PFS was defined as the time from the initiation of cetuximab treatment until disease progression or death. The duration of survival was calculated in months, while the survival data were censored at the time of the last follow-up.

### 2.4. Data Analysis

The quantitative data and patient characteristics were presented using descriptive statistics. The continuous variables were expressed as the mean, standard deviation, and a 95% confidence interval (CI) and compared using analysis of variance or Student’s *t*-test, whereas the categorical variables were expressed as frequencies and percentages and compared using the chi-square test. The survival curves were plotted using the Kaplan–Meier method, and the differences between the subgroups were assessed using the log-rank test. The Mann–Whitney U test was used to compare the differences in the treatment duration between the subgroups. All statistical analyses were performed using SPSS (version 24.0; IBM Corp, 2016. IBM SPSS Statistics for Windows, version 24.0; IBM Corp, Armonk, NY, USA), with statistical significance set at a *p*-value of <0.05.

## 3. Results

### 3.1. Characteristics of the Patients

This study retrospectively collected data from 67 patients with recurrent or metastatic head and neck cancer who underwent cetuximab treatment between 1 January 2020 and 31 May 2024. Table 1 shows the clinical characteristics of all patients. All 67 patients were male, with an initial diagnosis age ranging from 28 to 74 years. Fourteen patients (20.9%) were under the age of 45, 35 patients (52.2%) were between the ages of 46 and 60, and 18 patients (26.9%) were over the age of 61, indicating a median age of 54 years. The primary tumor location was in the oral cavity in 31 patients (46.3%), the pharynx in 32 patients (47.8%), and others in four patients (6.0%). Over 60% of the study population (*n* = 42) had stage IV disease, 13.4% (*n* = 9) had stage I disease, 17.9% (*n* = 12) had stage II disease, and 4.5% (*n* = 3) had stage III disease. The p16 status of some patients was evaluated, with four patients (6.0%) being positive and 19 patients (28.4%) being negative. A total of 29 patients (43.3%) had distant metastasis, 16 patients (23.9%) had both distant metastasis and recurrence, and 16 patients (23.9%) had only local recurrence. In prior treatments, 55 patients (82.1%) had OP, while 10 patients (14.9%) had only received CCRT without OP. Among those who had previously been treated with cisplatin, 27 patients (40.3%) had a treatment interval of more than six months, while 25 patients (37.3%) had an interval of less than six months. In subsequent lines of treatment, nine patients (13.4%) received IO therapy, whereas 58 patients (86.6%) did not. With regard to the treatment regimens, 25 patients (37.3%) were administered CPF4, 25 patients (37.3%) CPF2, and five patients (7.5%) CTPF.

When the cases were stratified based on patients’ treatment regimens, due to the small number of patients in the CTPF group (*n* = 5), the following comparison focused only on the clinical characteristics and outcomes between the CPF4 and CPF2 groups. No significant differences were observed between the CPF4 and CPF2 groups in terms of age, primary tumor location, stage, p16 status, cancer treatment history (whether OP was performed), time interval since the last use of cisplatin (whether less than six months), or the use of IO therapy. However, a significant difference was observed in the disease extent. Over 80% of patients with both recurrence and metastasis were administered CPF4, whereas only 30–40% of patients with local recurrence or distant metastasis were administered CPF4. Table 2 shows the detailed data.

### 3.2. Survival Analysis

**Figure 1:**
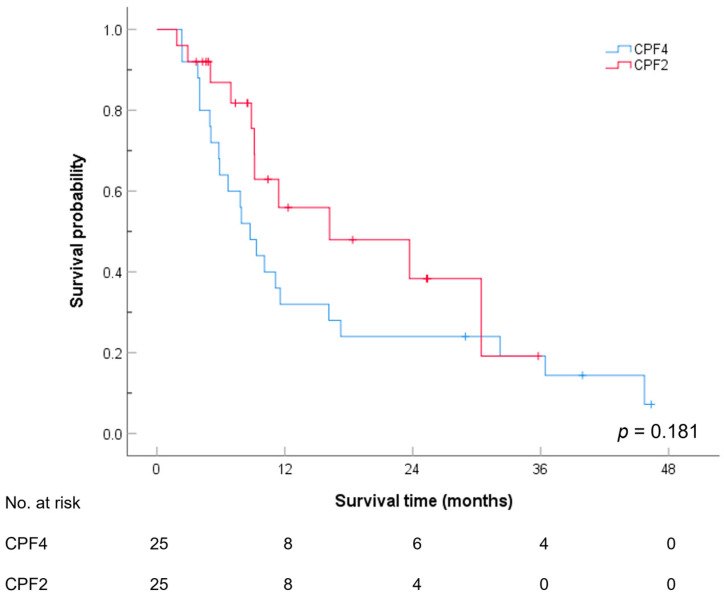
Overall survival stratified by the treatment regimens.

Figure 1 and Table 3 show the overall survival stratified by treatment regimens. Comparatively, no significant difference was observed in the overall survival between the CPF4 and CPF2 groups (*p*-value = 0.181), with the median overall survival being 8.7 months (95% CI, 6.3–11.2 months) for the CPF4 group and 16.2 months (95% CI, 0–33.7 months) for the CPF2 group. The 12-month survival rate was 32.0% (95% CI, 13.7–50.3%) for the CPF4 group and 55.9% (95% CI: 32.1–79.8%) for the CPF2 group. Moreover, the 24-month survival rate was 24.0% (95% CI: 7.3–40.7%) for the CPF4 group and 38.3% (95% CI: 12.2–64.5%) for the CPF2 group. The 36-month survival rate was 19.2% (95% CI: 3.4–35.0%) for the CPF4 group and 19.2% (95% CI: 0–48.8%) for the CPF2 group.

**Figure 2:**
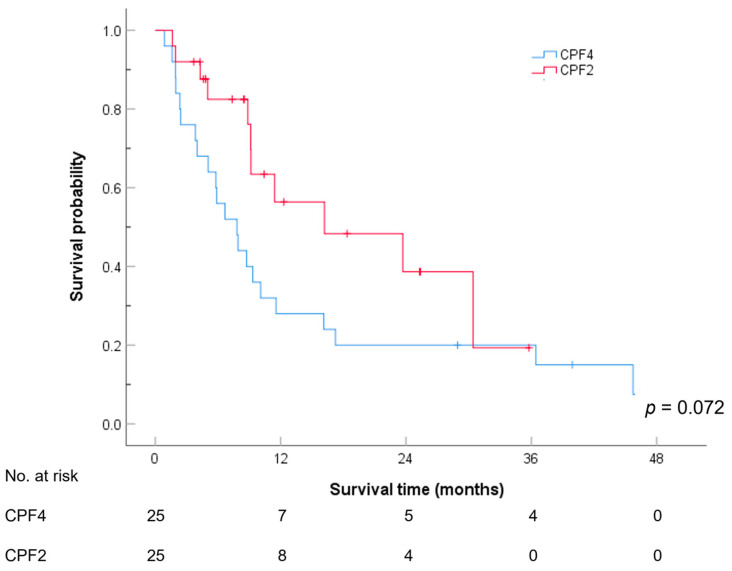
PFS stratified by the treatment regimens.

Figure 2 and Table 4 show the PFS for the CPF4 and CPF2 groups. No significant difference was observed in the PFS between the CPF4 and CPF2 groups (*p*-value = 0.072). The median PFS was 7.8 months (95% CI: 4.5–11.1 months) for the CPF4 group and 16.2 months (95% CI: 0–33.6 months) for the CPF2 group. The 12-month PFS rates were 28.0% (95% CI: 10.4–45.6%) and 56.4% (95% CI: 32.6–80.2%), respectively; the 24-month PFS rates were 20.0% (95% CI: 4.3–35.7%) and 38.7% (95% CI: 12.4–65.0%), respectively; and the 36-month PFS rates were 20.0% (95% CI: 4.3–35.7%) and 19.3% (95% CI: 0–49.2%), respectively.

**Figure 3:**
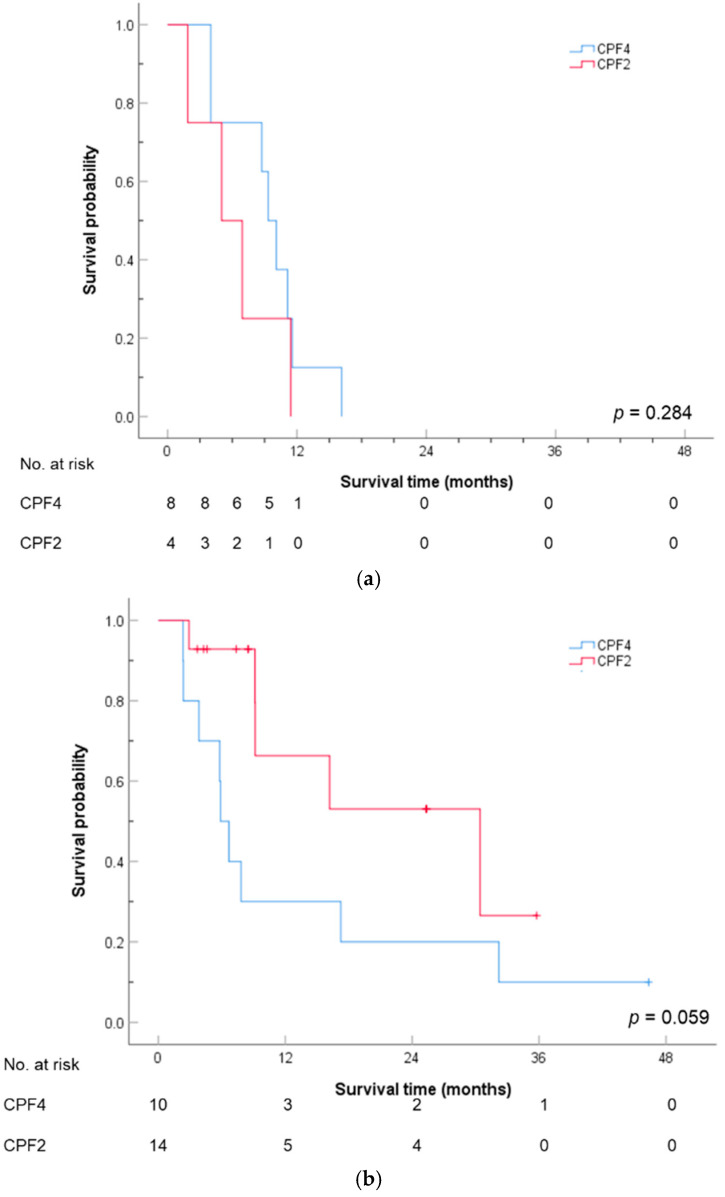

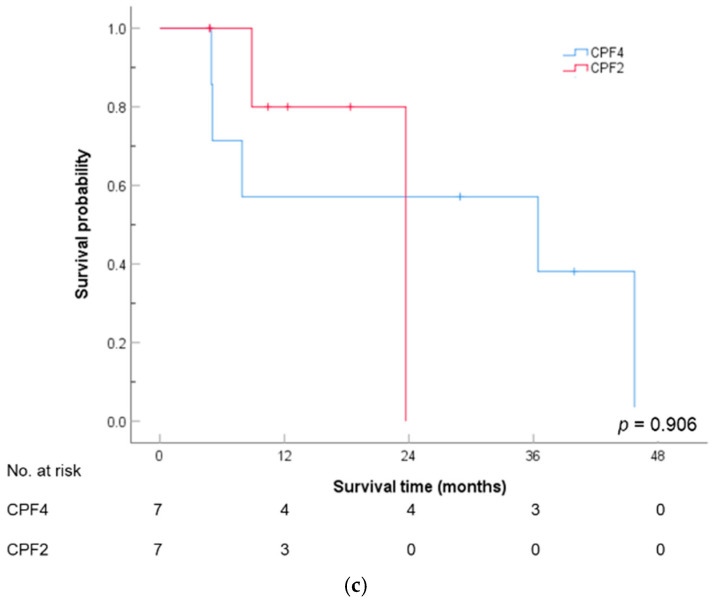
Overall survival curves by treatment regimens stratified by age group: (**a**) age ≤ 45 years; (**b**) 46–60 years; and (**c**) >60 years.

Table 5 shows the Cox regression analysis of the survival rates for potential factors. The univariate analysis revealed that younger individuals were associated with a higher risk of mortality. Specifically, the hazard ratio for the 46–60 age group was 0.32 times higher than that of individuals under 45 (95% CI: 0.15–0.67), while for the 61–75 age group, the hazard ratio was 0.27 times higher than that of those under 45 (95% CI: 0.11–0.66). Other factors, including location, stage, disease extent, p16 status, cancer treatment history (whether OP was performed), time interval since the last use of cisplatin, and IO usage, were not significantly associated with mortality risk. To account for the potential interactions between the covariates, the treatment regimen and age were included in the Cox regression model, as shown in Table 6. Although the results were not significant at the 5% significance level (α = 0.1), the patients aged 46–60 years who were administered CPF2 had a significantly lower risk—approximately 0.23 times—compared with those who were administered CPF4. Figure 3 shows the overall survival curves for the two treatment regimens across the three age groups (≤45, 46–60, >60 years). The results suggest that the impact of the treatment regimen on the survival time varied across age groups. In the 46–60 age group, the survival difference between CPF4 and CPF2 was significant (*p* = 0.059), indicating a potential survival advantage for CPF2 over CPF4, although no statistical significance (α = 0.05) was observed. In patients aged ≤45 and >60 years, no significant difference in the survival rates between the two treatment regimens was observed (*p*-values of 0.284 and 0.906, respectively).

**Figure 4:**
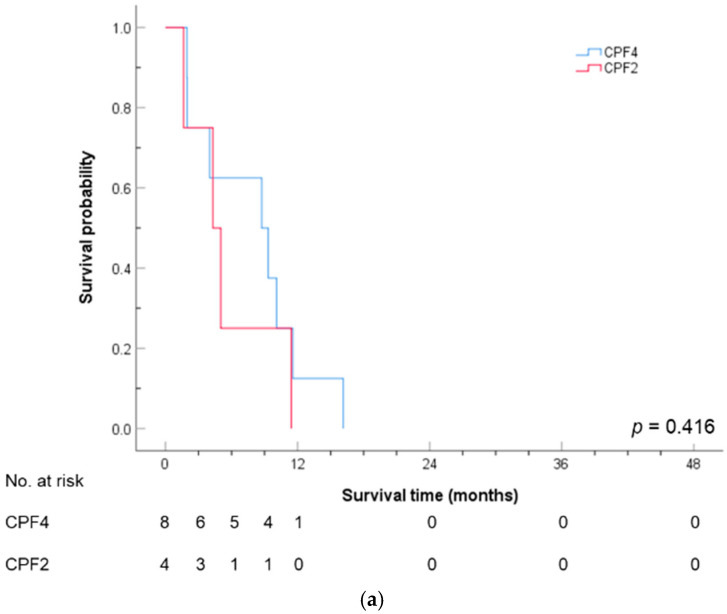

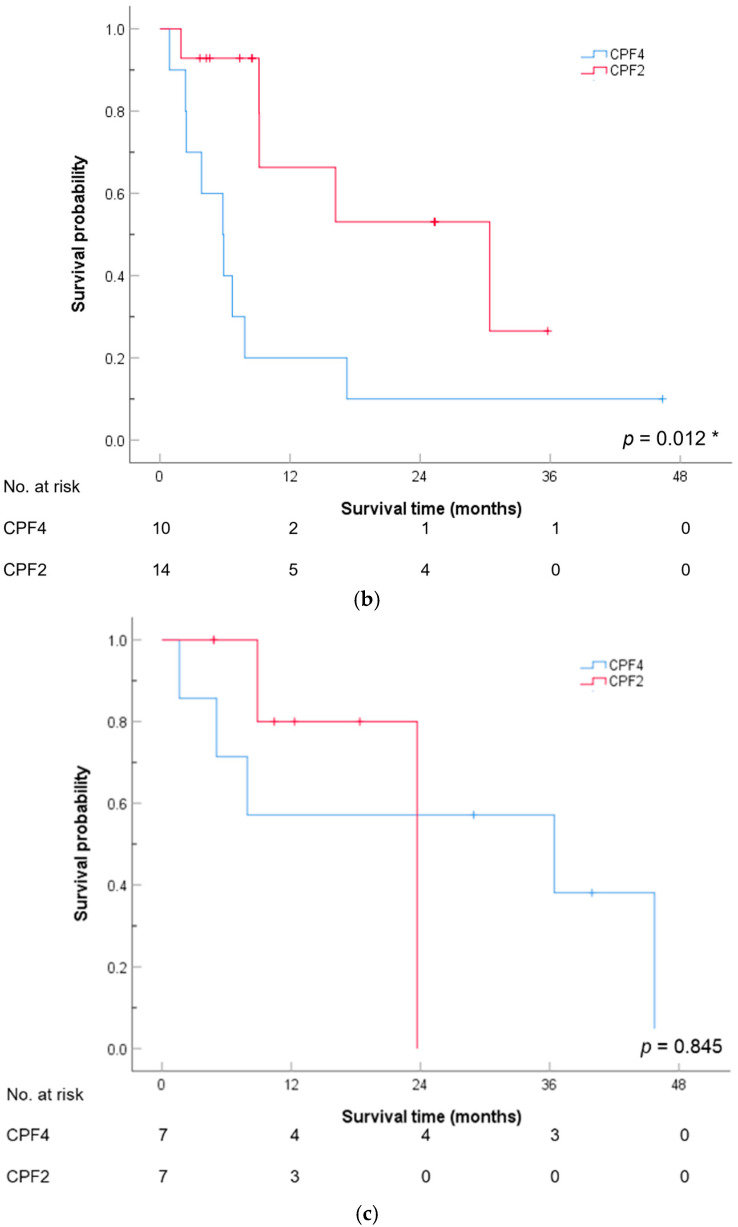
PFS curves by treatment regimens stratified by age group: (**a**) age ≤45 years; (**b**) 46–60 years; and (**c**) >60 years. * Statistically significant.

Similarly, Table 7 shows the Cox regression analysis of PFS for potential factors. Consistent with the overall survival results, the younger individuals were at a higher risk, with the 46–60 age group having a hazard ratio of 0.33 times higher than that of those under 45 (95% CI: 0.16–0.67), while the 61–75 age group had a hazard ratio of 0.26 times higher than that of those under 45 (95% CI: 0.11–0.63). While the difference in the risk between the two treatment regimens was not significant at α = 0.05 (*p*-value = 0.077), it became significant at α = 0.1, suggesting a possible advantage of CPF2 over CPF4 in the PFS. The hazard ratio for CPF2 was 0.51 higher than CPF4 (95% CI: 0.25–1.08). The interaction between the treatment regimen and age was taken into account, as shown in Table 8. Among the patients aged 46–60 years, CPF2 showed a significant PFS advantage over CPF4 (*p*-value = 0.049), with the hazard ratio for CPF2 being 0.19 times higher than that of CPF4 (95% CI: 0.04–0.99). Figure 4 shows the PFS curves for the two treatment regimens across the three age groups. In the 46–60 age group, CPF4 and CPF2 showed a significant difference in the PFS (*p*-value = 0.012). In contrast, for patients aged ≤45 and >60 years, no significant difference was observed in the PFS between the two treatment regimens (*p*-values of 0.416 and 0.845, respectively).

## 4. Discussion

This study aimed to investigate the effectiveness of cetuximab treatment in patients with recurrent or metastatic head and neck cancer at a hospital in Southern Taiwan. The clinical information of 67 patients with head and neck cancer who underwent cetuximab combination treatment between January 2020 and May 2024 was evaluated using retrospective observational methodology. The effects of different cetuximab combination regimens on patient survival were compared. Based on the findings, the CPF2 regimen may have improved the PFS in the 46–60 age group. Although CPF2 and CPF4 did not significantly vary in terms of overall survival rates, CPF2 showed a potential advantage over CPF4 at a significance threshold of α = 0.1. These findings emphasize the importance of patient age and treatment convenience in therapy selection. However, the improved PFS observed with CPF2 may be partially attributable to its longer treatment duration (median: 18.4 weeks vs. 8.1 weeks for CPF4). This highlights the need to balance treatment efficacy with patient-specific factors, including the ability to tolerate prolonged treatment.

In contrast to CPF4, which is administered once every four weeks, CPF2 is administered more frequently—once every two weeks. The higher frequency of CPF2 may result in more stable medication concentrations, which might slow down the progression of the illness. This is especially true for the 46–60 age group, which appears to have an advantage in the PFS. Furthermore, since CPF2 is administered on an outpatient basis, it lessens the requirement for hospitalization, thereby improving the quality of life of the patients. While CPF2 reduces hospitalization requirements and enhances patient quality of life, the extended treatment duration may impose additional time and financial burdens on patients. This therapeutic method may help minimize the financial burden on patients and maximize healthcare resources by lowering hospital bed utilization. Therefore, therapy selection should be individualized, considering both clinical efficacy and patient preferences.

In the present study, the patients administered cetuximab combined with cisplatin and 5-Fu (regardless of CPF4 or CPF2 group) had a median overall survival of 11.1 months (95% CI: 7.8–14.5 months) and a median PFS of 9.3 months (95% CI: 6.4–12.3 months), which were comparable to the findings from other randomized trials. The 2008 EXTREME trial, a phase III randomized study targeting recurrent or metastatic SCCHN, reported a median overall survival of 10.1 months (95% CI: 8.6–11.2 months) and a PFS of 5.6 months in patients who were treated with cisplatin and 5-Fu chemotherapy combined with cetuximab [14]. Similarly, a retrospective study in Portugal reported a median overall survival of 11 months (95% CI: 8.7–13.3 months) and a PFS of 8 months (95% CI: 6.1–9.9 months) [15]. In 2016, a retrospective observational study that was conducted across six head and neck cancer treatment centers in the Netherlands revealed that patients treated with platinum plus 5-Fu and cetuximab had a median overall survival of 6.7 months (95% CI: 4.4–8.9 months) and a PFS of 4.8 months (95% CI: 3.2–6.4 months) [16]. Moreover, a prospective observational study in India in the same year reported a median overall survival of 9.9 months (95% CI: 8.6–11.3 months) and a PFS of 5.3 months (95% CI: 4.5–6.1 months) [17]. In 2017, a single-center retrospective study from Austria reported that patients treated with the EXTREME regimen had a median overall survival of 8.4 months and a PFS of 4.8 months [18]. A 2018 multicenter retrospective observational study in Japan evaluating the effectiveness of cetuximab combined with platinum-based chemotherapy in patients with recurrent or metastatic oral squamous cell carcinoma reported a median overall survival of 12.1 months and a PFS of 7.8 months [19]. Another Japanese multicenter retrospective cohort study on 100 patients analyzed the real-world effectiveness of the EXTREME regimen as the first-line treatment for recurrent/metastatic SCCHN, indicating a median overall survival of 11 months and a PFS of 5 months [20]. A retrospective analysis in India in 2020 revealed that patients with recurrent/metastatic SCCHN who were treated with palliative chemotherapy combined with cetuximab had a median overall survival of 11.8 months and a PFS of 8.1 months [21]. Pontes et al. (2021) reported a median PFS of 7.1 months (95% CI: 5.6–8.6 months) and a median overall survival of 11.7 months (95% CI: 10.5–12.8 months) [22]. Similarly, Yazilitas et al. (2023) showed a median PFS of 7 months (95% CI: 5.9–8.1 months) and a median overall survival of 9.6 months (95% CI: 4.0–14.0 months), further supporting the consistency of these findings with other real-world studies [23].

The results indicated a survival advantage in the PFS for patients aged 46–60 years who were treated with the CPF2 regimen. This finding suggests that this age group may tolerate more frequent dosing schedules better or possess pharmacokinetic characteristics that are more favorable for CPF2 treatment. Bonner et al. (2010) reported that in patients undergoing radiotherapy, those younger than 65 years might benefit more from integrating cetuximab into radiotherapy compared with radiotherapy alone [24]. Similarly, a meta-analysis by Lacas et al. (2021) on SCCHN chemotherapy revealed that younger patients derive greater survival benefits from chemotherapy, with the positive impact diminishing with age [25]. A 2021 real-world retrospective study observed a trend toward improved OS in younger patients treated with platinum-based chemotherapy plus cetuximab, although the results did not reach statistical significance [22]. Yazilitas et al. (2023) analyzed the EXTREME regimen (chemotherapy plus cetuximab) in recurrent/metastatic SCCHN and reported a hazard ratio (age <65 vs. >65) of 1.172 (*p*-value = 0.658), indicating no significant age-related advantage [23]. These findings suggest age-related variability in treatment response, though further investigation is needed. In contrast, no significant differences in the overall survival or PFS were observed between the CPF2 and CPF4 regimens in other age groups, possibly due to smaller sample sizes limiting statistical power.

Although CPF2 has a PFS advantage for specific age groups, it failed to significantly prolong overall survival. This may suggest that cetuximab provides short-term benefits in delaying tumor progression but does not substantially impact overall survival. Similar findings have been observed in other cancer treatments. A 2015 Dutch study on recurrent or metastatic head and neck cancer revealed that while the treatment group administered cetuximab combined with platinum and 5-Fu had no overall survival advantage over the other treatment groups, the median PFS was significantly higher. This supports the potential role of cetuximab in short-term disease control [16]. In our study, no treatment discontinuation was attributed to adverse effects, highlighting that both CPF2 and CPF4 regimens were generally well-tolerated by patients, regardless of their differing schedules. Treatment cessation was primarily attributed to disease progression rather than toxicity-related complications. This finding underscores the importance of selecting treatment regimens that balance efficacy with tolerability to maintain patient quality of life. However, CPF4, with its shorter treatment duration (median: 8.1 weeks), may be more suitable for patients who prioritize minimizing treatment time and associated burdens. Although no significant differences in the survival outcomes were observed between CPF2 and CPF4, treatment convenience becomes an essential consideration. CPF4 requires hospitalization for administration every four weeks, whereas CPF2 can be administered biweekly on an outpatient basis. Outpatient treatment reduces the burden of hospitalization, minimizes the inconvenience associated with hospital stays, and optimizes the utilization of healthcare resources. Moreover, inpatient treatment increases the risk of healthcare-associated infections (HAI). Assefa and Amare (2022) reported that bacterial biofilms could form on solid surfaces and medical devices (e.g., ventilators and catheters), leading to HAIs such as ventilator-associated pneumonia and bloodstream infections [26]. These findings underscore the importance of outpatient treatment in minimizing infection risks associated with hospitalization and improving patient quality of life.

This study has several limitations. First, the sample size was relatively small, and the single-center retrospective design may limit the external validity of the findings. Second, the study population included only male patients, reflecting the gender distribution of head and neck cancer in Taiwan. This limitation prevents an exploration of potential gender differences in treatment responses. Third, although the study stratified patients into three age groups (≤45 years, 46–60 years, and ≥61 years), the limited sample size in each group may reduce the statistical power to detect significant differences in treatment responses across age groups. Fourth, although the study compared two treatment regimens (CPF2 and CPF4), the limited sample size for the third regimen (CTPF) precluded a robust analysis. Furthermore, the exclusion of other potential treatment regimens may limit the generalizability of the findings to broader clinical settings. Additionally, genetic or biomarker stratification, such as p16 status or EGFR expression levels, was not conducted, and these factors may influence the effectiveness of cetuximab treatment. Future research should consider conducting larger-scale, multicenter prospective studies that include genetic markers and molecular characteristics to confirm the effectiveness of cetuximab treatment and its applicability across different age groups.

## 5. Conclusions

The findings of this study suggest that CPF2 may offer a PFS advantage in patients aged 46–60. Although no significant difference in overall survival was observed between CPF2 and CPF4, there are important practical considerations in the treatment administration. CPF4 requires hospitalization for administration every four weeks, while CPF2 can be administered every two weeks on an outpatient basis. As inpatient treatment increases hospital visits, complicates medical procedures, and compromises the patients’ daily lives, CPF2 has a better advantage as an outpatient regimen. For some patients, outpatient treatment can reduce unnecessary hospitalization burdens, thereby enhancing their quality of life. However, CPF2’s longer treatment duration may impose additional time and financial burdens, highlighting the importance of balancing treatment duration with patient tolerance. Therefore, therapy selection should be individualized, considering both clinical efficacy and patient preferences.

## Figures and Tables

**Table 1:** Baseline characteristics of the patients.

Characteristic	Number of Patients (%)
Sex	
Female	0 (0)
Male	67 (100)
Age (Years)	
Median	54 Years
Range	28–74 Years
20–45	14 (20.9)
46–60	35 (52.2)
61–75	18 (26.9)
Primary Tumor Site	
Oral Cavity	31 (46.3)
Pharynx	32 (47.8)
Others	4 (6.0)
Initial Stage	
Stage 0	1 (1.5)
Stage I	9 (13.4)
Stage II	12 (17.9)
Stage III	3 (4.5)
Stage IV	42 (62.7)
p16 Status	
Negative	19 (28.4)
Positive	4 (6.0)
Unknown	44 (65.7)
Disease Extent	
Recurrent	16 (23.9)
Metastatic	29 (43.3)
Recurrent/Metastatic	16 (23.9)
Unknown	6 (9.0)
Cancer Treatment History	
OP	55 (82.1)
CCRT without OP	10 (14.9)
Unknown	2 (3.0)
Interval Cisplatin	
≤6 Months	25 (37.3)
>6 Months	27 (40.3)
Unknown	15 (22.4)
IO Therapy Used	
No	58 (86.6)
Yes	9 (13.4)
Treatment Regimens	
CPF4	25 (37.3)
CPF2	25 (37.3)
CTPF	5 (7.5)
Others	12 (17.9)

Abbreviations: OP, operation; CCRT, concurrent chemoradiotherapy; Interval_cisplatin, time interval since the last use of cisplatin; IO, immune-oncology; CPF4, cetuximab combined with cisplatin and 5-Fu, which was administered every four weeks during hospitalization; CPF2, cetuximab combined with cisplatin and 5-Fu, which was administered every two weeks as outpatient treatment; CTPF, cetuximab combined with docetaxel, cisplatin, and 5-Fu, which was administered every three to four weeks in the outpatient or inpatient setting.

**Table 2:** Characteristics of the patients based on the treatment regimens.

Variable	No. of Patients (%)		*p*-Value
	CPF4	CPF2	
Age (Years)			0.368
20–45	8 (66.7)	4 (33.3)	
46–60	10 (41.7)	14 (58.3)	
61–75	7 (50.0)	7 (50.0)	
Primary Tumor Site			0.396
Oral Cavity	11 (44.0)	14 (56.0)	
Pharynx	14 (58.3)	10 (41.7)	
Stage			>0.999
0, I, II, III	9 (47.4)	10 (52.6)	
IV	16 (51.6)	15 (48.4)	
p16 Status			0.497
Negative	9 (56.3)	7 (43.8)	
Positive	2 (100.0)	0 (0.0)	
Disease Extent			0.029 *
Recurrent	4 (33.3)	8 (66.7)	
Metastatic	9 (42.9)	12 (57.1)	
Recurrent/Metastatic	10 (83.3)	2 (16.7)	
Cancer Treatment History			0.667
OP	22 (51.2)	21 (48.8)	
CCRT without OP	2 (33.3)	4 (66.7)	
Interval Cisplatin			0.751
≤6 Months	8 (44.4)	10 (55.6)	
>6 Months	11 (52.4)	10 (47.6)	
IO Therapy Used			>0.999
No	21 (48.8)	22 (51.2)	
Yes	4 (57.1)	3 (42.9)	

Abbreviations: CPF4, cetuximab combined with cisplatin and 5-Fu, which was administered every four weeks during hospitalization; CPF2, cetuximab combined with cisplatin and 5-Fu, which was administered every two weeks as outpatient treatment; OP, operation; CCRT, concurrent chemoradiotherapy; Interval_cisplatin, time interval since the last use of cisplatin; IO, immune-oncology therapy. * Statistically significant.

**Table 3:** Summary of the overall survival stratified by the treatment regimens.

	CPF4	CPF2
Sample Size	25	25
No. of Deaths (%)	22 (88.0)	11 (44.0)
Median OS (Months) [95% CI]	8.7 [6.3–11.2]	16.2 [0–33.7]
12–Month OS Rate (%) [95% CI]	32.0 [13.7–50.3]	55.9 [32.1–79.8]
24–Month OS Rate (%) [95% CI]	24.0 [7.3–40.7]	38.3 [12.2–64.5]
36–Month OS Rate (%) [95% CI]	19.2 [3.4–35.0]	19.2 [0–48.8]

Abbreviations: CPF4, cetuximab combined with cisplatin and 5-Fu, which was administered every four weeks during hospitalization; CPF2, cetuximab combined with cisplatin and 5-Fu, which was administered every two weeks as outpatient treatment; OS, overall survival; CI, confidence interval.

**Table 4:** Summary of the PFS stratified by the treatment regimens.

	CPF4	CPF2
Sample Size	25	25
No. of Deaths (%)	22 (88.0)	11 (44.0)
Median PFS (Months) [95% CI]	7.8 [4.5–11.1]	16.2 [0–33.6]
12–Month PFS Rate (%) [95% CI]	28.0 [10.4–45.6]	56.4 [32.6–80.2]
24–Month PFS Rate (%) [95% CI]	20.0 [4.3–35.7]	38.7 [12.4–65.0]
36–Month PFS Rate (%) [95% CI]	20.0 [4.3–35.7]	19.3 [0–49.2]

Abbreviations: CPF4, cetuximab combined with cisplatin and 5-Fu, which was administered every four weeks during hospitalization; CPF2, cetuximab combined with cisplatin and 5-Fu, which was administered every two weeks as outpatient treatment; PFS, progression-free survival; CI, confidence interval.

**Table 5:** Univariate analysis of the overall survival rates.

Variable	Number of Patients (%)	Hazard Ratio (95% CI)	*p*-Value
Age (Years)			
20–45	14 (20.9)	1	
46–60	35 (52.2)	0.32 (0.15–0.67)	0.002 *
61–75	18 (26.9)	0.27 (0.11–0.66)	0.004 *
Primary Tumor Site			
Oral Cavity	31 (46.3)	1	
Pharynx	32 (47.8)	0.70 (0.37–1.33)	0.271
Stage			
0–III	25 (37.3)	1	
IV	42 (62.7)	1.20 (0.65–2.24)	0.558
p16 Status			
Negative	19 (28.4)	1	
Positive	4 (6.0)	1.98 (0.51–7.66)	0.325
Disease Extent			
Recurrent	16 (23.9)	1	
Metastatic	29 (43.3)	1.36 (0.61–3.01)	0.453
Recurrent/Metastatic	16 (23.9)	0.74 (0.29–1.86)	0.523
Cancer Treatment History			
OP	55 (82.1)	1	
CCRT without OP	10 (14.9)	1.32 (0.51–3.41)	0.560
Interval Cisplatin			
≤6 Months	25 (37.3)	1	
>6 Months	27 (40.3)	1.02 (0.51–2.02)	0.955
IO Therapy Used			
No	58 (86.6)	1	
Yes	9 (13.4)	1.04 (0.49–2.18)	0.925
Treatment Regimen			
CPF4	25 (37.3)	1	
CPF2	25 (37.3)	0.61 (0.29–1.27)	0.186

Abbreviations: CI, confidence interval; OP, operation; CCRT, concurrent chemoradiotherapy; time interval since the last use of cisplatin; IO, immune-oncology; CPF4, cetuximab combined with cisplatin and 5-Fu, which was administered every four weeks during hospitalization; CPF2, cetuximab combined with cisplatin and 5-Fu, which was administered every two weeks as outpatient treatment. * Statistically significant.

**Table 6:** Results for the interaction models of the overall survival.

Variable	Hazard Ratio ^1^ (95% CI ^2^)	*p*-Value
46–60 Years (vs. 20–45 Years)	0.76 (0.27–2.12)	0.599
61–75 Years (vs. 20–45 Years)	0.33 (0.10–1.12)	0.075
Treatment Regimen: CPF2 (vs. CPF4)	1.70 (0.51–5.72)	0.388
Interaction: 46–60 Years × CPF2	0.23 (0.04–1.21)	0.083
Interaction: 61–75 Years × CPF2	0.42 (0.05–3.39)	0.419

^1^ A hazard ratio of >1 indicates an increased risk of mortality; a hazard ratio of <1 indicates decreased risk. Interaction terms assess whether the effect of CPF2 compared to CPF4 differs across age groups. ^2^ Confidence interval.

**Table 7:** Univariate analysis of the PFS rate.

Variable	Number of Patients (%)	Hazard Ratio (95% CI)	*p*-Value
Age (Years)			
20–45	14 (20.9)	1	
46–60	35 (52.2)	0.33 (0.16–0.67)	0.002 *
61–75	18 (26.9)	0.26 (0.11–0.63)	0.003 *
Primary Tumor Site			
Oral Cavity	31 (46.3)	1	
Pharynx	32 (47.8)	0.66 (0.35–1.25)	0.200
Stage			
0–III	25 (37.3)	1	
IV	42 (62.7)	1.31 (0.70–2.43)	0.399
p16 Status			
Negative	19 (28.4)	1	
Positive	4 (6.0)	1.92 (0.49–7.43)	0.347
Disease Extent			
Recurrent	16 (23.9)	1	
Metastatic	29 (43.3)	1.44 (0.65–3.19)	0.371
Recurrent/Metastatic	16 (23.9)	0.94 (0.38–2.34)	0.897
Cancer Treatment History			
OP	55 (82.1)	1	
CCRT without OP	10 (14.9)	1.41 (0.55–3.63)	0.475
Interval Cisplatin			
≤6 Months	25 (37.3)	1	
>6 Months	27 (40.3)	0.85 (0.43–1.68)	0.630
IO Used			
No	58 (86.6)	1	
Yes	9 (13.4)	1.43 (0.68–3.01)	0.351
Treatment Regimen			
CPF4	25 (37.3)	1	
CPF2	25 (37.3)	0.51 (0.25–1.08)	0.077

Abbreviations: CI, confidence interval; OP, operation; CCRT, concurrent chemoradiotherapy; time interval since the last use of cisplatin; IO, immune-oncology therapy; CPF4, cetuximab combined with cisplatin and 5-Fu, which was administered every four weeks during hospitalization; CPF2, cetuximab combined with cisplatin and 5-Fu, which was administered every two weeks as outpatient treatment. * Statistically significant.

**Table 8:** Results for the interaction models of the PFS.

Variable	Hazard Ratio ^1^ (95% CI ^2^)	*p*-Value
46–60 Years (vs. 20–45 Years)	0.86 (0.32–2.36)	0.777
61–75 Years (vs. 20–45 Years)	0.32 (0.10–1.07)	0.064
Treatment Regimen: CPF2 (vs. CPF4)	1.57 (0.47–5.27)	0.467
Interaction: 46–60 Years × CPF2	0.19 (0.04–0.99)	0.049 *
Interaction: 61–75 Years × CPF2	0.40 (0.05–3.19)	0.389

^1^ A hazard ratio of >1 indicates an increased risk of mortality; a hazard ratio of <1 indicates decreased risk. Interaction terms assess whether the effect of CPF2 compared to CPF4 differs across age groups. ^2^ Confidence interval. * Statistically significant.

## Data Availability

The data presented in this study are not publicly available due to ethical and legal restrictions. Access to the anonymized datasets may be granted upon reasonable request to the corresponding author, pending approval from the Institutional Review Board of E-Da Hospital.
